# Single Nucleotide Variations in *CLCN6* Identified in Patients with Benign Partial Epilepsies in Infancy and/or Febrile Seizures

**DOI:** 10.1371/journal.pone.0118946

**Published:** 2015-03-20

**Authors:** Toshiyuki Yamamoto, Keiko Shimojima, Noriko Sangu, Yuta Komoike, Atsushi Ishii, Shinpei Abe, Shintaro Yamashita, Katsumi Imai, Tetsuo Kubota, Tatsuya Fukasawa, Tohru Okanishi, Hideo Enoki, Takuya Tanabe, Akira Saito, Toru Furukawa, Toshiaki Shimizu, Carol J. Milligan, Steven Petrou, Sarah E. Heron, Leanne M. Dibbens, Shinichi Hirose, Akihisa Okumura

**Affiliations:** 1 Tokyo Women’s Medical University Institute for Integrated Medical Sciences, Tokyo, Japan; 2 Department of Hygiene and Public Health I, Tokyo Women’s Medical University, Tokyo, Japan; 3 Department of Pediatrics, Fukuoka University Faculty of Medicine, Fukuoka, Japan; 4 Department of Pediatrics, Juntendo University Faculty of Medicine, Tokyo, Japan; 5 Department of Pediatrics, Juntendo Nerima Hospital, Tokyo, Japan; 6 National Epilepsy Center, Shizuoka Institute of Epilepsy and Neurological Disorders, Shizuoka, Japan; 7 Department of Pediatrics, Anjo Kosei Hospital, Anjo, Japan; 8 Department of Child Neurology, Seirei Hamamatsu General Hospital, Hamamatsu, Japan; 9 Tanabe Monbayashi Child Clinic, Hirakata, Japan; 10 StaGen Co., Ltd., Tokyo, Japan; 11 Florey Neuroscience Institute, Melbourne Brain Centre, The University of Melbourne, Melbourne, Victoria, Australia; 12 Epilepsy Research Program, School of Pharmacy and Medical Sciences, University of South Australia, Adelaide, South Australia, Australia; Osaka University Graduate School of Medicine, JAPAN

## Abstract

Nucleotide alterations in the gene encoding proline-rich transmembrane protein 2 (*PRRT2*) have been identified in most patients with benign partial epilepsies in infancy (BPEI)/benign familial infantile epilepsy (BFIE). However, not all patients harbor these *PRRT2* mutations, indicating the involvement of genes other than *PRRT2*. In this study, we performed whole exome sequencing analysis for a large family affected with *PRRT2*-unrelated BPEI. We identified a non-synonymous single nucleotide variation (SNV) in the voltage-sensitive chloride channel 6 gene (*CLCN6*). A cohort study of 48 BPEI patients without *PRRT2* mutations revealed a different *CLCN6* SNV in a patient, his sibling and his father who had a history of febrile seizures (FS) but not BPEI. Another study of 48 patients with FS identified an additional SNV in *CLCN6*. Chloride channels (CLCs) are involved in a multitude of physiologic processes and some members of the CLC family have been linked to inherited diseases. However, a phenotypic correlation has not been confirmed for *CLCN6*. Although we could not detect significant biological effects linked to the identified *CLCN6* SNVs, further studies should investigate potential *CLCN6* variants that may underlie the genetic susceptibility to convulsive disorders.

## Introduction

Benign partial epilepsy in infancy (BPEI) is an epileptic syndrome described by Watanabe and Okumura [1]. BPEI is analogous to benign familial infantile epilepsy (BFIE) according to the revised terminology for organization of seizures and epilepsies [2]. The clinical features of BPEI include the onset of epilepsy during 3 to 10 months of age, clustering seizures, absence of abnormalities in electroencephalogram (EEG) or neuroimaging, favorable outcome of seizure control, and normal neurodevelopment [3]. Additionally, about 40% affected children have a family history of BPEI [3]. Some BPEI patients demonstrate paroxysmal kinesigenic dyskinesia (PKD), suggesting an overlap between BPEI and infantile convulsions and choreoathetosis syndrome (ICCA). We have also shown that approximately 10% children with BPEI experience convulsions associated with mild gastroenteritis [3].

Recently, Chen et al. (2011) identified mutations in the gene encoding proline-rich transmembrane protein 2 (*PRRT2*) by whole exome sequencing analysis of eight Chinese families affected by autosomal-dominant PKD [4]. Subsequently, Heron et al. (2012) detected five different *PRRT2* mutations in 14 of 17 families affected by BPEI and in five of six families affected by ICCA [5]. These findings indicate that *PRRT2* is one of the major genes related to BPEI/BFIE and ICCA. However, not all BPEI patients harbor *PRRT2* mutations. In our study, mutated *PRRT2* was detected in about half of the Japanese BPEI patients, indicating the existence of other BPEI genes in the Japanese population.

In this study, we performed genomic analyses to identify additional genes involved in BPEI development.

## Materials and Methods

This study was approved by the ethical committee of Tokyo Women’s Medical University (registration #206). Written informed consent was obtained from all patients or their legal guardians.

### Subjects

Blood samples and clinical information were collected on patients afflicted with BPEI, convulsions with mild gastroenteritis, and febrile seizures (FS). We defined BPEI as epilepsy meeting all of the following conditions: (A) clinical diagnosis of focal seizures and/or secondary generalized seizures; (B) normal psychomotor development and neurological findings prior to seizure onset; (C) normal interictal EEG; (D) normal neuroimaging findings; and (E) seizure onset at 3–12 months of age [3,6]. All samples from patients with BPEI have been previously analyzed by nucleotide sequencing of *PRRT2* coding regions [7]. Patients’ clinical histories, with regard to seizure/convulsion episodes, were based on interviews of family members. FS definition was based on at least one seizure incident associated with pyrexia over 38°C.

### Whole exome sequencing

**Fig 1:**
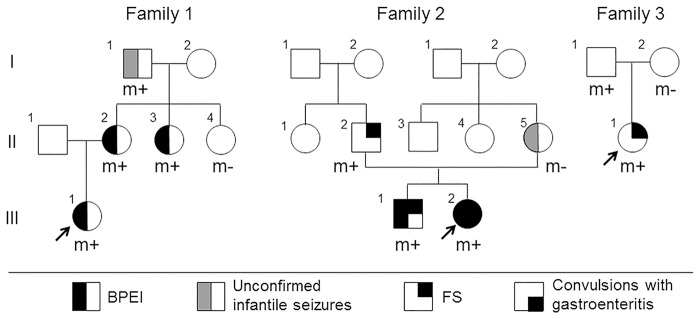
Family trees of three families harboring *CLCN6* variants. *CLCN6* variant-positive members are presented as (m+), and *CLCN6* variant-negative members are presented as (m-). Arrows indicate the proband in the family.

Whole exome sequencing was performed for a Japanese family covering three generations (Family 1) using the Agilent SureSelect Human All Exon Capture kit (Agilent Technologies, Santa Clara, CA) and pair-end sequencing on a SOLiD3 system (Life Technologies, Foster City, CA), as previously described [8]. Genomic DNA was isolated from blood samples of Family 1 members. Extracted results of the affected members of Family 1 (I-1, II-2, II-3, and III-1) were compared with that of the BPEI-unaffected member (II-4), used as a negative control (Fig. 1).

For prioritization, we focused only on non-synonymous variants, splice acceptor and donor site mutations, and frameshift insertion/deletions (indel) (S1 Fig.). We excluded the candidate variants that were located in segmental duplication regions and listed in the 1000 Genomes Project (http://www.1000genomes.org/) and dbSNP 132 (http://www.ncbi.nlm.nih.gov/projects/SNP/). Because we assumed an autosomal dominant trait in Family 1, the variants shared by all the affected family members but not detected in the unaffected member were selected. Then, select non-synonymous variants were tested for mutational effects using amino acid substitution prediction tools, PolyPhen-2 [9] (http://genetics.bwh.harvard.edu/pph2/) and SIFT [10] (http://sift.jcvi.org/). Extracted variants were finally evaluated by Sanger sequencing as described elsewhere [11] to determine whether they segregated with the disease in this family.

### Candidate gene validation

After selecting the candidate gene with a possible relationship to BPEI, we performed a cohort study to identify nucleotide alterations in the gene. Cohort 1 included 48 BPEI patients negative for *PRRT2* mutations and six children with a history of convulsions associated with mild gastroenteritis. Cohort 2 consisted of 48 unrelated patients with FS. All coding exons of *CLCN6* were analyzed by standard Sanger sequencing. Primer information is available in S1 Table. One hundred samples from healthy Japanese individuals were also used. Statistical analysis was performed using Fisher’s exact test.

### Cell biological analysis

To confirm the pathological significance of the non-synonymous single nucleotide variants (SNVs) identified in this study, we compared the expression patterns and biological functions of the identified SNV-containing *CLCN6* with those of wild type *CLCN6* in COS-1 cells transfected with the respective expression plasmids. For this purpose, we constructed a plasmid encoding human wild type *CLCN6* complementary DNA (cDNA) and introduced two different SNVs (G250S and R319Q) into it.

Human Brain Total RNA purchased from Clontech (#636530; Mountain View, CA) was reverse-transcribed to cDNA using the SuperScript VILO cDNA Synthesis Kit (Life Technologies) according to the manufacturer’s instruction. Then, *CLCN6* transcripts were amplified by PCR using the specific primers 5’-GGATCCGCCACCATGGCGGGGTGCAGGGGGTC-3’ and 5’-GGATCCTTAAACTCGCCAAAGTTCAG-3’, and the amplicons were cloned into the pGEM-T vector (Promega, Madison, WI). Twenty clones were established and genotyped by Sanger sequencing using T7 and Sp6 primers. Transcript variant 1–3 was selected and its full-length cDNA was subcloned into the pFLAG-CMVTM-2 expression vector containing the promoter-regulatory region of human cytomegalovirus upstream of the FLAG epitope (E7398; Sigma-Aldrich, St. Louis, MO, USA). Because R319Q is located at the *CLCN6* 3’-terminus, we used a 5’-UTR fusion of FLAG. Finally, the *CLCN6* transcript variant 1–3 mutants carrying G250S and R319Q were created using the KOD-Plus-Mutagenesis Kit (SMK-101; TOYOBO, Osaka, Japan). The expression plasmids encoding either wild type *CLCN6* or *CLCN6* transcript variant 1–3 containing the two SNVs were introduced into COS-1 cells using Lipofectamine 2000 Reagent (Life Technologies). The subcellular localization of the recombinant proteins was analyzed by immunofluorescence using antibodies against FLAG (F7425, Sigma-Aldrich), protein disulfide isomerase (PDI) as an ER marker (RL90; Abcam, Cambridge, UK), and DAPI (P36931; Life Technologies). Cell lysates were analyzed by western blot using anti-FLAG antibody as previously described [11].

A patch clamp assay was performed to evaluate the physiological effects of the recombinant proteins (Supplemental Information).

## Results

### Molecular analyses

**Fig 2:**
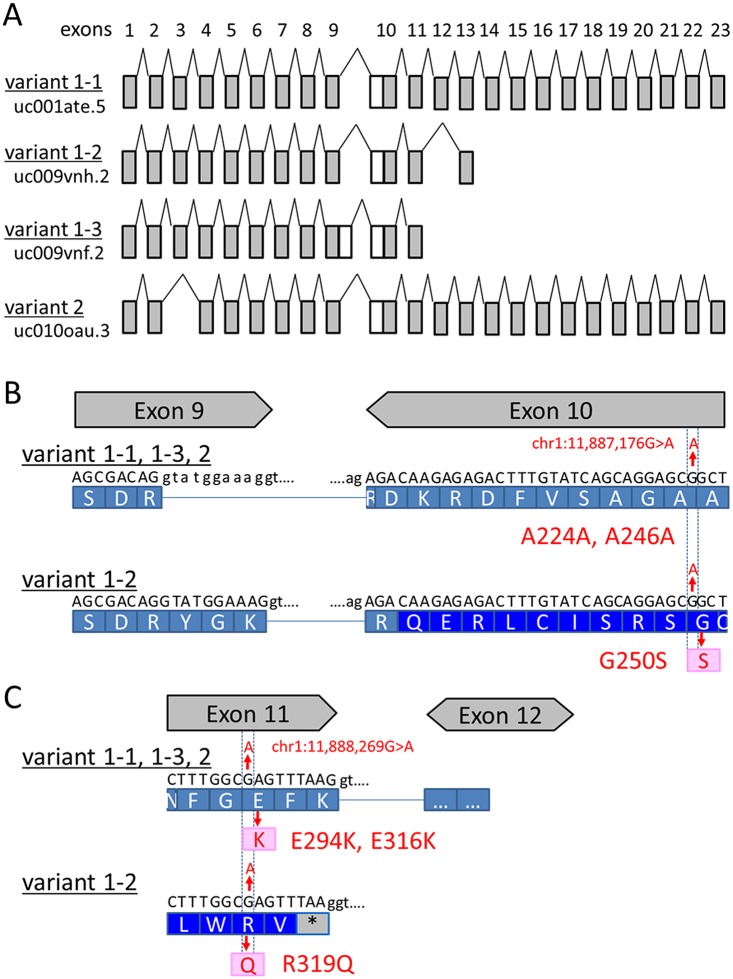
Exon usage and location of *CLCN6* transcript variants. (A) Exon usage of four coding transcript variants. (B) Schematic representation of the locations of the SNVs identified in this study for each *CLCN6* transcript variant. Two exon-intron boundaries are highlighted to clarify the complicated exon usage in the region.

**Fig 3:**
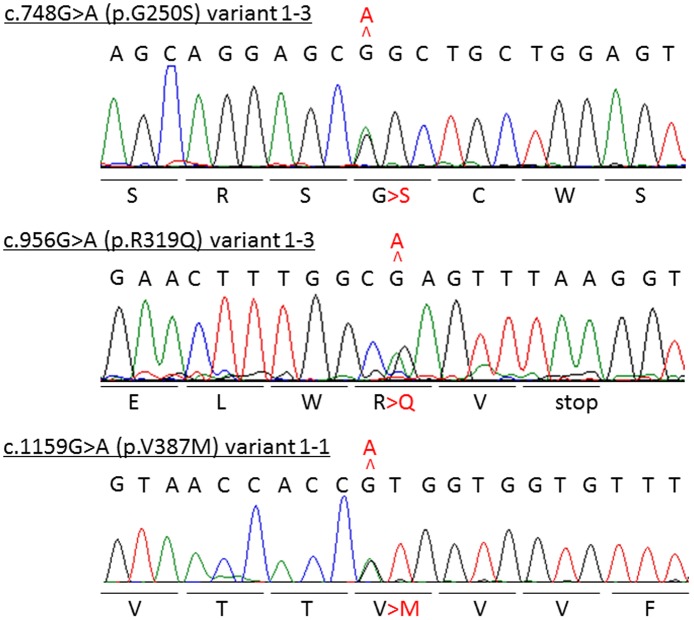
Electropherograms of the identified *CLCN6* variants confirmed by Sanger sequencing. Identified variants are shown in red.

Whole exome sequencing produced an average of 1.76×10^8^ sequence reads aligned to the reference genome (85.6% of which was properly mapped) with a mean coverage of 68.7 (S2 Table). The variants were filtered according to the flow chart shown in S1 Fig.; five SNVs and one insertion in six genes were selected as the candidate genes (Table 1). Among them, an SNV in the *CLCN6* coding region, chr1:11,887,176G>A was of particular interest because of its functional relevance and previously published linkage data [12]. Seven transcript variants are listed in the UCSC genome browser database (https://genome.ucsc.edu/), and the selected SNV has been identified in one of these transcript variants (transcript variant 1–3 [uc009vnf.2]: c.748G>A [p.G250S]) (Fig. 2B, Table 2). Sanger sequencing identified this SNV (Fig. 3) in all affected members of Family 1 members but not in the unaffected member (Fig. 1; II-4), confirming its segregation with the disease. None of the Family 1 members had mutations in *PRRT2*. The identified SNV was absent in 100 normal Japanese individuals.

**Table 1:** Candidate genes selected by filtering.

Chromosome	Position*	Region	Gene name	Function	Reference	Alteration	PolyPhen2	SIFT
chr1	11,887,176	exon	*CLCN6*	non-synonymous SNV	G	A	0.619445	0.02
chr9	140,069,578	exon	*ANAPC2*	non-synonymous SNV	A	G	0.999	0
chr11	102,738,797	exon	*MMP12*	frameshift insertion	-	T	NA	NA
chr12	6,952,360	exon	*GNB3*	non-synonymous SNV	G	T	0.999	0
chr17	74,276,523	exon	*QRICH2*	non-synonymous SNV	T	C	0.98	0
chr22	50,927,689	exon	*MIOX*	non-synonymous SNV	G	A	1	0

*, genomic positions are referred to build19; SNV, single nucleotide variation; NA, not applicable

**Table 2:** *CLCN6* transcript variants and identified variants in this study.

Transcript variants	RefSeq annotation number	UCSC annotation number	Genome position	Length of amino acid	Coding exon counts	Type of RNA	SNVs identified in this study		
							1st SNV	2nd SNV	3rd SNV
Transcript variant 1–1	NM_001286	uc001ate.5	chr1:11,866,153–11,903,201	870	23	mRNA	c.738G>A (p.A246A)	c.946G>A (p.E316K)	c.1159G>A (p.V387M)
Transcript variant 1–2	NM_001286	uc009vnh.2	chr1:11,866,153–11,889,379	354	12	mRNA	c.738G>A (p.A246A)	c.946G>A (p.E316K)	NA
Transcript variant 1–3	NM_001286	uc009vnf.2	chr1:11,866,153–11,888,276	321	11	mRNA	c.748G>A (p.G250S)	c.956G>A (p.R319Q)	NA
Transcript variant 2	NM_001256959	uc010oau.3	chr1:11,866,153–11,903,201	848	22	mRNA	c.672G>A (p.A224A)	c.880G>A (p.E294K)	NA
Transcript variant 3–1	NR_046428	uc010oat.3	chr1:11,866,153–11,903,201	260	23	non-coding	NI	NI	NI
Transcript variant 3–2	NR_046428	uc009vng.2	chr1:11,866,153–11,888,276	309	11	non-coding	NI	NI	NI
Transcript variant 3–3	NR_046428	uc009vne.2	chr1:11,866,153–11,876,844	85	3	non-coding	NI	NI	NI

SNV, single nucleotide variant; NA, not affected; NI, not indicated

Next, we performed a cohort study for *CLCN6* in 48 BPEI patients without *PRRT2* mutations and six patients who had convulsions associated with mild gastroenteritis. The Cohort 1 study identified a non-synonymous SNV in exon 10, c.956G>A (p.R319Q) in the members of Family 2 (Figs. 1 and 3). This SNV affected all four coding transcript variants by non-synonymous alteration R>Q or E>K (Fig. 2B, Table 2). Among the 100 normal Japanese controls, this SNV was identified in one individual. Although this SNV was not detected in the mother with a history of unconfirmed infantile seizures (Fig. 1; II-5), it was found in the father who had FS, suggesting a possible linkage between *CLCN6* and FS. The Cohort 2 study of 48 unrelated FS patients identified another non-synonymous SNV, c.1159G>A (p.V387M) in exon 3 among Family 3 members. Although this SNV was not identified in 100 normal Japanese individuals, it is included in dbSNP build 138 as rs201349073, with an allele frequency of 0.092% (2/2179).

Thus, the incidence of *CLCN6* SNVs was 3% (3/102) in patients with BPEI and/or FS, which was higher than that in normal controls (1/100). However, statistical analysis by Fisher’s exact test showed a p-value of 0.25, which did not suggest a significant difference.

### Mutagenesis assay

**Fig 4:**
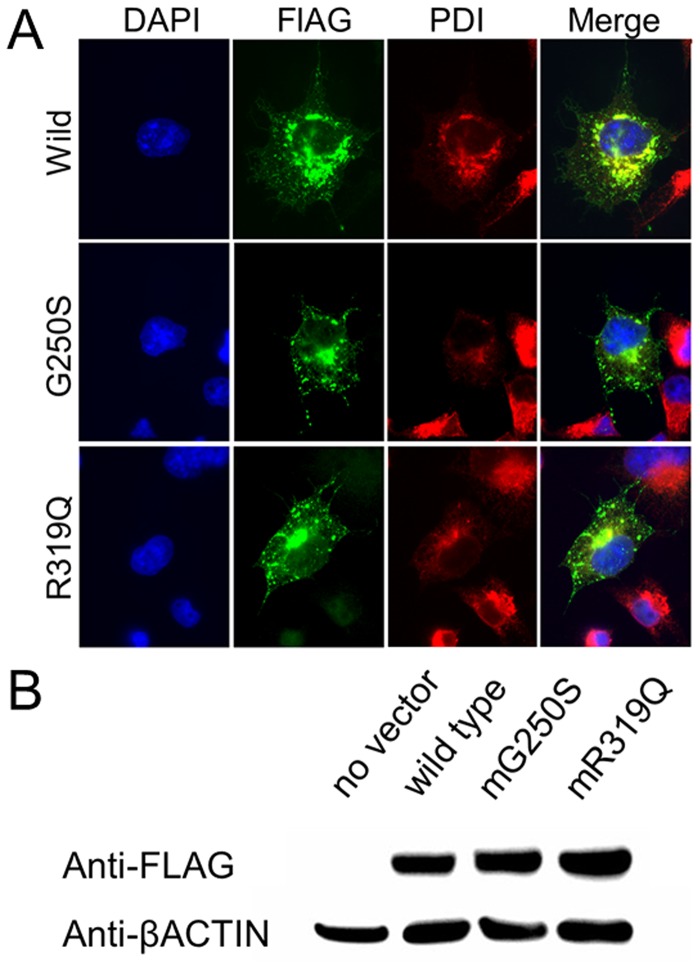
In vitro functional evaluation of SNVs effects. (A) Immunofluorescence staining of COS1 cells transfected with SNV-harboring *CLCN6* variants. Protein disulfide isomerase (PDI) is used as marker of the endoplasmic reticulum (ER). FLAG-tagged CLCN6 is merged with PDI, indicating CLCN6 localization in the ER. (B) Western blotting analysis of cell lysates shows no difference in CLCN6 expression.

Among the 20 clones produced by subcloning of the reverse transcription-PCR amplicons, one had sequence corresponding to that of transcript variant 1–3. The expression of FLAG-tagged CLCN6 was successfully confirmed in the transfected cells (Fig. 4A), where it was predominantly co-localized with PDI in the endoplasmic reticulum (ER). However, no differences in subcellular localization were detected between the wild type and mutants (Fig. 4A), and no differences in expression levels were observed by western blot (Fig. 4B). Patch-clamp analysis too did not reveal any significant functional difference between the wild type and mutant variants (S2 Fig.).

### Clinical information

In Family 1 (Fig. 1), the proband (III-1) was a girl who was first presented with unprovoked seizures at the age of 8 months and was subsequently diagnosed with BPEI. Her mother (II-2), maternal aunt (II-3), and maternal grandfather (I-1) had a history of infantile seizures, but the other aunt (II-4) had no seizure history. The proband (III-2) of Family 2 was a girl diagnosed with BPEI (Fig. 1). Her elder brother (III-1) also had BPEI, while her father (II-2) had experienced one simple FS. The detailed case histories are available in the S1 Supporting Information.

## Discussion

In this study, whole exome sequencing for a three-generation family with *PRRT2* mutation-negative BPEI showed six SNVs: five non-synonymous alterations and one frameshift change in six genes (Table 1). Among them, *CLCN6* located on 1p36.22 was considered the most promising candidate based on previous findings suggesting a linkage between BFIE and the 1p36.12-p35.1 locus [12]. *CLCN6* belongs to a family of chloride channels (CLCs) involved in a multitude of physiologic processes ranging from basal cellular functions such as cell volume control and acidification of intracellular vesicles to more specialized mechanisms [13], including regulation of electrical excitability, transepithelial transport, electroneutrality, and ionic homeostasis [14]. In mammals, the CLC family comprises nine members that differ in biophysical properties, cellular compartmentalization, and tissue distribution [15]. Among them, four members have been associated with inherited disorders. The mutations in the voltage-sensitive chloride channel genes *CNCNKB*, *CLCN1*, *CLCN5*, and *CLCN7* have been linked to Bartter syndrome, myotonia congenita, Dent disease, and osteopetrosis, respectively [16,17,18,19]. Furthermore, variations in *CLCN1*, *CLCN2*, and *CLCN4* have been reported in patients with idiopathic epilepsy and epileptic encephalopathy [20,21,22,23]; however, the association of some variants with disease pathogenesis is still controversial [24].

Although *CLCN6* and *CLCN7* form a distinct branch of the CLC gene family, sharing 45% sequence homology with each other [15], CLCN6 is the least well-characterized mammalian CLC protein [25]. *CLCN6* mRNA is expressed in many tissues, including the brain and kidney [15], and CLCN6 has been reported to co-localize with the markers of ER or endosomes [26,27]. Knock-out of *CLCN6* in mice did not result in increased lethality or produce a strong phenotype [25], but moderate neuronal pathology, resembling that in mild forms of human neuronal ceroid lipofuscinosis (NCL), has been observed [28]. However, genetic analysis of 75 NCL patients identified only two heterozygous mutations in *CLCN6* [25].

On the other hand, a genome-wide association study (GWAS), conducted to identify potential genetic modifiers of cardiac hormonal response, showed a link between the N-terminal signal peptide of pro-B-type natriuretic peptide (NT-proBNP) and *CLCN6* variants. However, it did not exclude the possibility that the identified *CLCN6* variants may simply be a marker for unobserved causal variants in the neighboring gene locus [29]. Thus, phenotypic correlation of *CLCN6* with human diseases has not been confirmed.

In this study, we tested the hypothesis that *CLCN6* is another gene responsible for BPEI onset by analyzing samples from BPEI patients without *PRRT2* mutations by whole-exome sequencing. Because both *CLCN6* SNVs identified in Families 1 and 2 commonly affected transcript variant 1–3, the functional relevance of these SNVs was analyzed in vitro; however, no definite difference was observed between the cells expressing wild type and mutant variants. Therefore, we do not have sufficient evidence to suggest that these *CLCN6* SNVs have a significant pathological impact.

The SNV identified in Family 2 was shared with the parent who had FS, but not with the other parent who had infantile seizures. There is no contradiction in this finding, given that 15% of BPEI patients have FS [30]. The SNV identified in Family 2 was also detected in one of the 100 control samples (1%; 1/100). We subsequently examined a relationship between *CLCN6* SNVs and FS in a cohort of FS patients and identified the third SNV in a patient who had a single FS attack. The third SNV identified in Family 3 was listed in the SNV database but with a very low incidence of 0.1%. Overall, the data indicate that the incidence of *CLCN6* SNVs in patients with BPEI and/or FS was 3% (3/102), which was not significantly higher than in the general population (1%). Because FS is a relatively common condition, occurring in 2–5% of infants in Europe and North America and in 6–9% of infants in Japan [31], the existence of the same *CLCN6* SNVs in the general population should not be a reason of discounting the relationship between *CLCN6* SNVs and BPEI and/or FS.

There are many mutations in the ion-channel genes that show low penetrance in segregation [32]. Indeed, *PRRT2* mutations are often shared with non-phenotypic carriers in families with a history of BPEI [7], suggesting that SNV-related clinical effects would not be significant in episodic disorders. Given that, in this study, *CLCN6* SNVs have been identified in patients with BPEI or FS, such SNVs may not be BPEI-specific but could have a milder association with convulsive disorders including BPEI and FS. The second SNV identified in Family 2 members with or without BPEI/FS produces a non-synonymous substitution in all *CLCN6* transcript variants; however, the first SNV identified in Family 1 members with BPEI results in a non-synonymous substitution only for transcript variant 1–3. Meanwhile, the third SNV identified in Family 3 members with FS produces a non-synonymous substitution only for the other transcript variants. These results suggest that SNVs in different *CLCN6* transcript variants may be related to distinct phenotypes (i.e., BPEI and/or FS). Alternatively, it may be possible that the observed variants generally shift genetic predisposition toward seizures.

This study was aimed at identifying another gene responsible for BPEI, but SNVs in *CLCN6* were found in only a small proportion of BPEI patients. Thus, the data are inconclusive. Recent massive parallel sequencing for patients with sporadic epilepsy of unknown etiology identified SNVs in the chloride channel genes, *CLCN1* and *CLCN2* [33], suggesting an association of CLCs with epilepsy. In that study, *CLCN6* variants made up a small proportion of the patients but were not present in the controls (detailed results unavailable). Therefore, there is still a possibility that *CLCN6* variants are related to genetic susceptibility for convulsive disorders such as BPEI and FS. Further investigation is required to test this possibility.

## Supporting Information

S1 Fig Filtering steps in the selection of the variants extracted by whole exome sequencing. (PDF)

S2 Fig Wild-type and mutant hCLCN6 currents recorded in *Xenopus oocytes*. **(A)** Averaged current-voltage relationships for the oocytes injected with wild-type (WT, solid line; n = 10), G250S (dotted line; n = 8), or R318Q (dashed line; n = 6) *CLCN6* cDNA or water (H2O dot-dash line; n = 8). Oocytes were held at-20mV and stepped from-100mV to 100 mV for 800 msec every 10 sec in 20 mV increments. (B) Average peak currents at 100 mV for WT (n = 10), G250S (n = 8), R319Q (n = 6), and H2O (n = 8).
(PDF)

S1 Supporting Information Supplemental information. Supplemental methods and results are included.
(PDF)

S1 Table Primers used for *CLCN6* Sanger sequencing. (PDF)

S2 Table The result of the mapping of whole-exome sequencing data for family 1. (PDF)
