# Supplementary figures and images for: Single Nucleotide Variations in CLCN6 Identified in Patients with Benign Partial Epilepsies in Infancy and/or Febrile Seizures
Source: PLoS One. 2015 Mar 20;10(3):e0118946. doi: 10.1371/journal.pone.0118946 (PMC4368117; doi:10.1371/journal.pone.0118946)

## Supplemental Figure S1. Filtering steps of the variants extracted by whole exome sequencing.

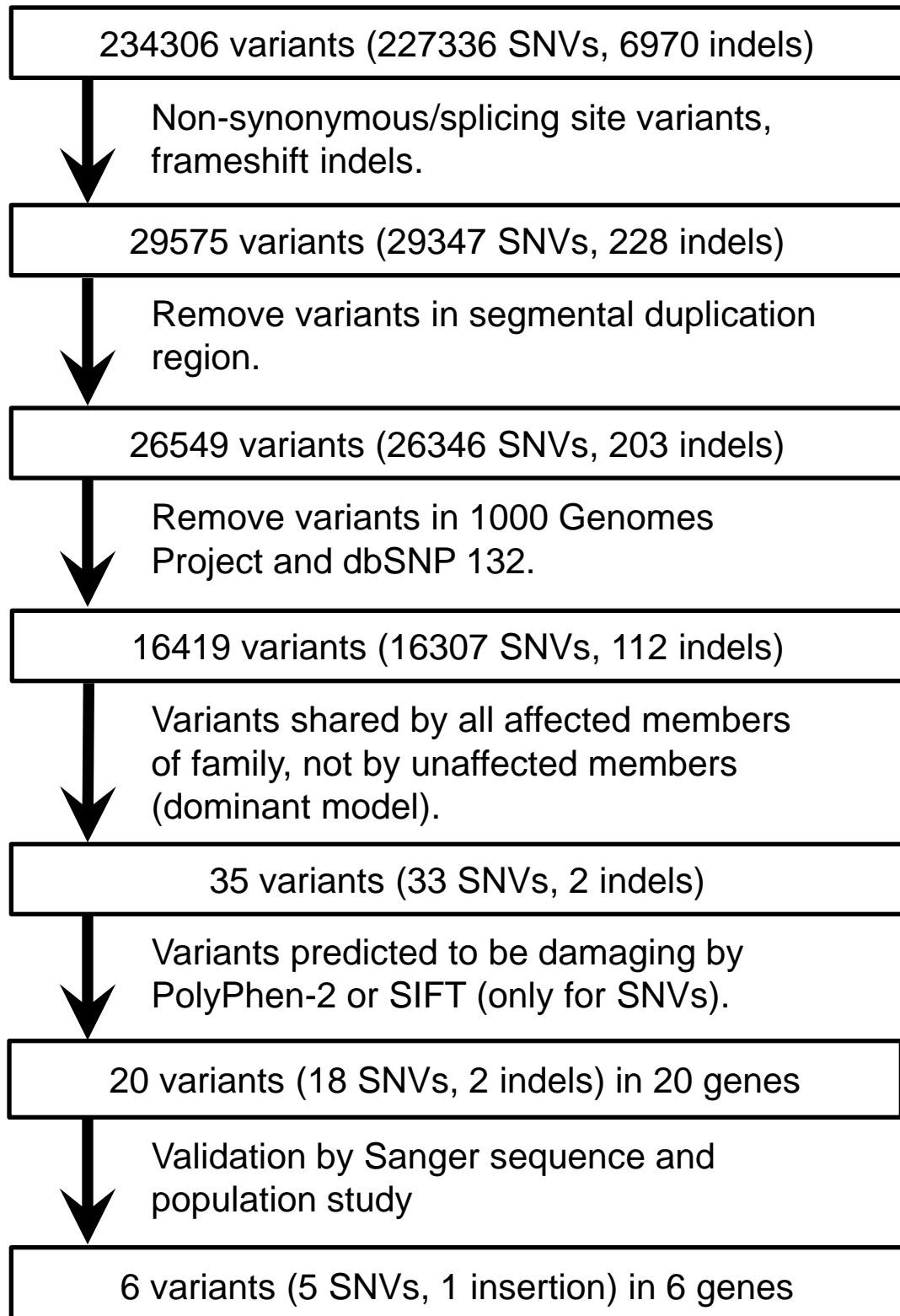

Supplement: S1 Fig — (PDF) [file pone.0118946.s001.pdf]
