# Supplementary material for: Single Nucleotide Variations in CLCN6 Identified in Patients with Benign Partial Epilepsies in Infancy and/or Febrile Seizures
Source: PLoS One. 2015 Mar 20;10(3):e0118946. doi: 10.1371/journal.pone.0118946 (PMC4368117; doi:10.1371/journal.pone.0118946)
Supplement: S2 Fig — (A) Averaged current-voltage relationships for the oocytes injected with wild-type (WT, solid line; n = 10), G250S (dotted line; n = 8), or R318Q (dashed line; n = 6) CLCN6 cDNA or water (H2O dot-dash line; n = 8). Oocytes were held at-20mV and stepped from-100mV to 100 mV for 800 msec every 10 sec in 20 mV increments. (B) Average peak currents at 100 mV for WT (n = 10), G250S (n = 8), R319Q (n = 6), and H2O (n = 8). (PDF) [file pone.0118946.s002.pdf]

Supplemental Figure S2. Wild-type and mutant *hCLCN6* currents recorded in *Xenopus oocytes*

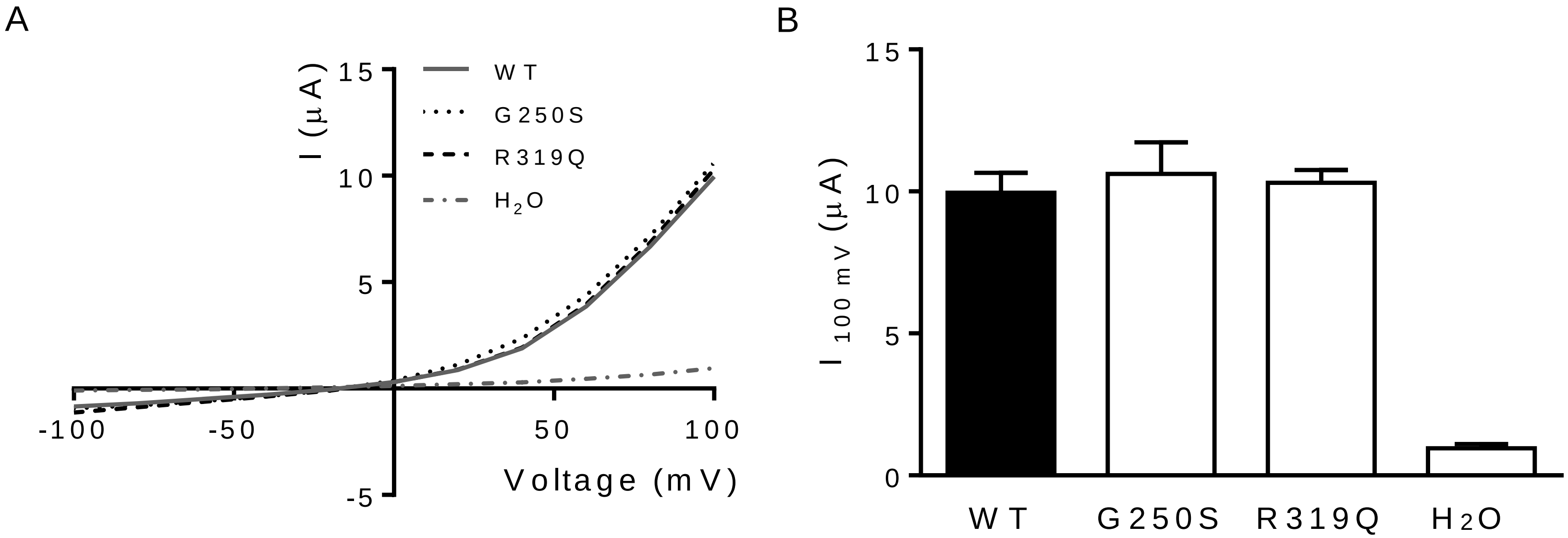

(A) Averaged current-voltage relationships for the oocytes injected with wild-type (WT, solid line; n = 10), G250S (dotted line; n = 8), or R319Q (dashed line; n = 6) CLCN6 cDNA or water (H_2O dot-dash line; n = 8). Oocytes were held at -20 mV and stepped from -100 mV to 100 mV for 800 msec every 10 sec in 20 mV increments. (B) Average peak currents at 100 mV for WT (n = 10), G250S (n = 8), R319Q (n = 6), and H_2O (n = 8).
