# Supplementary material for: Single Nucleotide Variations in CLCN6 Identified in Patients with Benign Partial Epilepsies in Infancy and/or Febrile Seizures
Source: PLoS One. 2015 Mar 20;10(3):e0118946. doi: 10.1371/journal.pone.0118946 (PMC4368117; doi:10.1371/journal.pone.0118946)
Supplement: S1 Supporting Information — Supplemental methods and results are included. (PDF) [file pone.0118946.s003.pdf]

Supplemental Information

Supplemental methods

Expression of human *CLCN6* in *Xenopus* oocytes

CLCN6 mutants (c.748G>A [p.G250S] and c.965G>A [p.R319Q]) were created as described in the Materials and Methods section in the main text. The expression plasmids were constructed by inserting the wild-type (WT) and mutant human *CLCN6* coding sequence into the pGEMHE vector [1] at the *Hin*dIII restriction site and were verified by Sanger sequencing. The constructs were cut with *Nhe*I and transcribed *in vitro* (mMessage mMachine, Ambion, Austin, TX, USA). *Xenopus* oocytes (Dumont stage V or VI) were surgically removed from *Xenopus laevis* and prepared as described previously [2]. Oocytes were stored in ND96 solution at 17 °C. Capped (c)RNA was injected into each oocyte (30 ng in 50 nl) using Roboinject1 System (Multi Channel Systems, Reutlingen, Germany).

Electrophysiology

All electrophysiological experiments were performed at room temperature (20–22 °C) on *Xenopus* oocytes subjected to a temperature increase (24 °C for 3 h) prior to the electrophysiological analysis. After 36–48 h of *CLCN6* injection, two electrode voltage clamp recordings were performed using the Roboocyte2 System (Multi Channel Systems, Reutlingen, Germany). Oocytes were impaled with electrodes containing 1.5 M K-acetate and 0.5 M KCl, held at -20 mV, and perfused with ND96 bath solution containing 96 mM NaCl, 2 mM KCl, 1.8 mM $CaCl_2$, 1 mM $MgCl_2$, and 5 mM HEPES, pH 7.5. To record expressed membrane currents, the oocytes were held at -20 mV, and 800-ms test depolarisation was applied every 10 s in 20 mV increments from -100 mV to 100 mV at the recording frequency of 1 kHz. The current produced by WT and mutant *CLCN6* were always measured contemporaneously using the same batch of oocytes to exclude the possibility of batch-to-batch variation in expression. Electrophysiological data were acquired and analyzed using AxoGraph (AxoGraph Scientific, Sydney, Australia). Statistical analysis was performed using GraphPad Prism 6 (GraphPad Software, La Jolla, CA, USA). The data are presented as the mean ± S.E.M and Student’s *t*-test was used to evaluate statistical significance.

Supplemental results

Detailed clinical characteristics of the patients

In family 1 (Figure 1), the proband (III-1) was a girl first presented to us because of unprovoked seizures at 8 months of age. She was the only offspring of unrelated parents. Perinatal and past history, psychomotor development, and physical and neurological examinations were unremarkable. EEG and brain magnetic resonance imaging (MRI) revealed no abnormalities. The girl was diagnosed with BPEI and treated with carbamazepine since 9 months of age. Thereafter, she had sporadic seizures until 21 months. During the last follow-up at 26 months, she demonstrated normal development, although formal intelligence quotient (IQ) had not been evaluated. Her mother (II-2) had unprovoked seizures at 9 months of age. Although she had five seizures in 2 days, no seizures were observed thereafter and no antiepileptic drugs were administered. One of the maternal aunts of the proband (II-3) also had one unprovoked seizure at 2 months of age. In addition, the maternal grandfather (I-1) had some unprovoked infantile seizures, although the detailed information was not available. The other aunt of the proband (II-4) had no seizure history. None of the family 1 members had any delay in psychomotor development or other neurological, psychiatric, or behavioral disorders.

The proband (III-2) of family 2 was a girl who had the first unprovoked seizure at 11 months of age (Figure 1). She was the second offspring of unrelated parents. Her perinatal and past history was unremarkable. The girl achieved normal psychomotor development, and physical and neurological examinations were normal. No abnormal findings were observed in EEG and brain MRI. Although she had four unprovoked seizures within 5 days, she did not receive antiepileptic treatment and no unprovoked seizures were observed thereafter. She had brief generalized convulsions provoked by fever at 13 and 15 months. In addition, one generalized convulsion was observed at 30 months due to gastroenteritis without pyrexia. Her elder brother (III-1) also had a history of unprovoked seizures since 8 months of age. His seizures were controlled by phenobarbital for 4 months, and the drug was discontinued; however, a recurrence of unprovoked seizures was observed thereafter. Therefore, carbamazepine was administered since 12 months of age; however, he showed hypersensitivity to the drug 12 days after treatment initiation. Carbamazepine was discontinued and thereafter no antiepileptic drug was administrated. Since then, he did not have unprovoked seizures as documented at the last follow-up at 6 years and 8 months. He had had three brief generalized convulsions provoked by fever since 11 months, but no convulsion associated with gastroenteritis was observed. The father (II-2) had one simple FS at 2

years and the mother (II-5) had three unprovoked seizures in one day at around 1.5 years of age. None of family 2 members had delay in psychomotor development or other neurological, psychiatric, or behavioral disorders.

Proband from family 3 (II-1) had a single FS episode (Figure 1). Both parents had no history of FS and none of family 3 members had delays in psychomotor development or other neurological, psychiatric, or behavioral disorders.

***CLCN6* transcripts affected by the identified single nucleotide variants**

Seven different alternatively spliced transcript variants of *CLCN6* were revealed using the UCSC genome browser (Figure 2A and Table 2) (three types of transcript variant 3 are recognized as non-coding). The first SNV identified in family 1, chr1:11,887,176G>A, is located in exon 10 and presumed to result in a non-synonymous substitution, G>S, in transcript variant 1-3 (c.748G>A [p.G250S]), while producing a synonymous change, A>A, in transcript variant 1-1/1-2 (c.738G>A [p.A246A]) and transcript variant 2 (c.672G>A [p.A224A]) (Figure 2B and 3). The second SNV identified in family 2, chr1:11,888,268 G>A, is located in exon 11 and presumed to result in two types of non-synonymous substitutions: 1) E>K in transcript variant 1-1/1-2 (c.946G>A [p.E316K]) and transcript variant 2 (c.880G>A [p.E294K]), and 2) R>Q in transcript variant 1-3 (c.965G>A [p.R319Q]) (Figure 2C and 3). The third SNV identified in family 3 is located in exon 13 and presumed to result in a non-synonymous substitution, V>M, in transcript variant 1-1 (c.1159G>A [p.V387M] (Figure 3). Because the first and second SNVs, G250S and R319Q, presumes to result in amino acid changes in transcript variant 1-3, we have selected this variant as the target of the functional study.

Electrophysiological consequences of CLCN6 mutants

The electrophysiological properties of the *CLCN6* mutants (p.G250S, p.R319Q) identified in BPEI patients were analyzed in *Xenopus* oocytes. The WT and mutant *CLCN6* channels produced voltage-activated currents, while water-injected controls did not differ from the uninjected oocytes with only small contributions of endogenous currents. The shapes of averaged current-voltage (*I/V*) curves calculated for the WT and mutant *CLCN6* channels were not different. The hCLCN6-induced currents were outwardly rectifying (Supplementary Figure S2). Peak current amplitude measured at 100 mV showed no significant difference between the current produced by the WT and mutant CLCN6 proteins (Supplementary Figure S2).

References

1. Liman ER, Tytgat J, Hess P (1992) Subunit stoichiometry of a mammalian K+ channel determined by construction of multimeric cDNAs. Neuron 9: 861-871.
2. Petrou S, Ugur M, Drummond RM, Singer JJ, Walsh JV, Jr. (1997) P2X7 purinoceptor expression in Xenopus oocytes is not sufficient to produce a pore-forming P2Z-like phenotype. FEBS Lett 411: 339-345.
