# Supplementary material for: Single Nucleotide Variations in CLCN6 Identified in Patients with Benign Partial Epilepsies in Infancy and/or Febrile Seizures
Source: PLoS One. 2015 Mar 20;10(3):e0118946. doi: 10.1371/journal.pone.0118946 (PMC4368117; doi:10.1371/journal.pone.0118946)
Supplement: S1 Table — (PDF) [file pone.0118946.s004.pdf]

**Table S1. Primers used for *CLCN6* Sanger sequencing**

| | | Forward | | | Reverse | | Predicted PCR sizes |
|---|---|---|---|---|---|---|---|
| exon 1 | 5'- | AGCCACTCCTGGTCTCAGTC | -3' | 5'- | GTAGACGCTTCGAGAGCCC | -3' | 365 |
| exon 2 | 5'- | TTTAGAAGCTAGCCACTGATTTC | -3' | 5'- | AATCATAATGCTTCGGCCTG | -3' | 228 |
| exon 3 | 5'- | TCCACACGTTGAGTAGCAGG | -3' | 5'- | TGCCTGTATCGCATGAAAAG | -3' | 286 |
| exon 4 | 5'- | GCCAAGATGTATTTCTTCCCC | -3' | 5'- | ACCAGGCCAGGGCTTTC | -3' | 210 |
| exon 5 | 5'- | GATGCCTTCAAGAGGAGCAC | -3' | 5'- | GAATAGCTAATAAGCGGCCC | -3' | 269 |
| exon 6 | 5'- | CTGCACCCTCTCAAGTGATG | -3' | 5'- | AACAAGAGGCCACTTTCTGC | -3' | 304 |
| exon 7 | 5'- | CCAGATTGTTGAGGGTAAGCC | -3' | 5'- | GGCCCTGCAGTCCAAATC | -3' | 294 |
| exon 8 | 5'- | TTCAGTTGTCACGAGGAAGG | -3' | 5'- | ATTTGGCCCAGTGTAACCAG | -3' | 234 |
| exon 9 | 5'- | AGTCCTAGGCTGCCCCTG | -3' | 5'- | GGGATACTGCCAGGCTAGAG | -3' | 270 |
| exon 10 | 5'- | CAGAAACCACCTTTTGGGG | -3' | 5'- | GTACCGAGTAAGGGACACCC | -3' | 267 |
| exon 11 | 5'- | AGGAGGAAGGGTTGGGAG | -3' | 5'- | CTGCTCATTTCTCTCTGGTCC | -3' | 270 |
| exon 12 | 5'- | CTCTTGCTTCTGTCTTCTCTTC | -3' | 5'- | CTCCTCGGGAGAGATGGCG | -3' | 333 |
| exon 13 | 5'- | CTGCTGTGAATGCTGTTTGGAG | -3' | 5'- | GGAAGGATGAACCCAACATATC | -3' | 350 |
| exon 14 | 5'- | TGGACCCGGCTGGAGACATC | -3' | 5'- | ACACCAATCACCCACGATTG | -3' | 273 |
| exon 15/16 | 5'- | GGAACTGGTATGGGGTGTGG | -3' | 5'- | GTCCATTTGGCCACCTGAAAC | -3' | 713 |
| exon 17 | 5'- | CTGTCAGGCTCAGGGCCAC | -3' | 5'- | GTTACTGTTTCCTACCCTAATAAC | -3' | 262 |
| exon 18 | 5'- | AGGGCTGGAGAGCCAGCAC | -3' | 5'- | GATGAGAACAGTGCCAACTTCC | -3' | 400 |
| exon 19 | 5'- | CATTTGAACCACCAGCCTGAG | -3' | 5'- | GAAACTCAGGAGCCACCTCAC | -3' | 415 |
| exon 20 | 5'- | GGCTCAGATGTTGTGAGGTGG | -3' | 5'- | CCTATGGCACTGTTCACGGC | -3' | 375 |
| exon 21/22 | 5'- | TGGGCATATTCAGGCATCAAGG | -3' | 5'- | ACGGAAGCCGAGCAGGGTTC | -3' | 573 |
| exon 23 | 5'- | TCAAGCTGTGTCTGGCTGTGC | -3' | 5'- | GTGTCCGATGACTCCCGGC | -3' | 355 |

All primers are designed in the flanking intronic regions with at least 50 bp distance from exon/intron boundaries.
