# Supplementary material for: Single Nucleotide Variations in CLCN6 Identified in Patients with Benign Partial Epilepsies in Infancy and/or Febrile Seizures
Source: PLoS One. 2015 Mar 20;10(3):e0118946. doi: 10.1371/journal.pone.0118946 (PMC4368117; doi:10.1371/journal.pone.0118946)
Supplement: S2 Table — (PDF) [file pone.0118946.s005.pdf]

Table S2. The result of the mapping of whole exome sequencing data for family 1

Sample ID	I-1	II-2	II-3	II-4	III-1	Average
Mapped reads	180,881,812	211,837,042	165,146,156	166,414,396	158,612,804	181,069,852
Properly mapped reads	154,903,712	178,276,062	141,274,362	145,747,992	135,367,788	155,050,532
Total bases mapped	9,208,978,584	9,846,484,671	8,655,706,841	8,696,717,122	9,669,779,910	9,101,971,805
Total bases uniquely mapped (w/o duplicate reads)	4,930,351,738	6,352,622,514	6,001,427,793	5,189,537,228	5,095,590,495	5,618,484,818
Total bases mapping to target*1	2,409,199,406	4,094,923,264	2,451,038,966	3,198,637,153	3,323,511,309	3,038,449,697
Mean target*1 coverage	53.2	91.3	54.2	71.0	73.9	67.4
% target*1 bases with ≥10X coverage	86.85%	85.76%	85.10%	85.47%	85.21%	85.80%
Mean target*2 coverage	56.0	96.9	57.0	75.4	78.3	71.3
% target*2 bases with ≥10X coverage	84.21%	83.39%	82.83%	82.79%	83.25%	83.30%

*1: SureSelect bait, *2: RefSeq CDS
